# Risk and risk factors for disability pension among patients with treatment resistant depression– a matched cohort study

**DOI:** 10.1186/s12888-020-02642-9

**Published:** 2020-05-13

**Authors:** Heidi Taipale, Johan Reutfors, Antti Tanskanen, Lena Brandt, Jari Tiihonen, Allitia DiBernardo, Ellenor Mittendorfer-Rutz, Philip Brenner

**Affiliations:** 1 Department of Clinical Neuroscience, Division of Insurance Medicine, Karolinska Institutet, Stockholm, Sweden; 2 Department of Forensic Psychiatry, Niuvanniemi Hospital, University of Eastern Finland, Kuopio, Finland; 3 School of Pharmacy, University of Eastern Finland, Kuopio, Finland; 4 Centre for Pharmacoepidemiology, Department of Medicine Solna, Karolinska Institutet, Karolinska University Hospital, Stockholm, Sweden; 5 Janssen Research & Development, LLC, Titusville, NJ USA

**Keywords:** Major depressive disorder, Disability pension, Treatment resistant depression, Epidemiology, Antidepressants

## Abstract

**Background:**

Treatment resistant depression (TRD) is common among patients with depression, and is associated with clinical and functional disability. However, the risk and risk factors for being granted disability pension (DP) among patients with TRD have not been investigated.

**Methods:**

All antidepressant initiators in Sweden with a diagnosis of depression in specialized care were identified in nationwide registers 2006–2013 and followed regarding treatment trials. TRD was defined as the start of a third sequential trial. Patients with TRD who were not on DP (*N* = 3204) were matched by age, sex, history of depression, calendar year, and time for treatment start with 3204 comparators with depression and ongoing antidepressant treatment. A proportional Cox Regression was performed with DP as outcome, adjusted for various sociodemographic and clinical covariates.

**Results:**

Compared to the comparison cohort, TRD was associated with a doubled risk for all-cause DP (aHR 2.07; 95%CI 1.83–2.35), DP due to depression (2.28; 1.82–2.85) and to any mental disorder (2.24; 1.95–2.57) but not due to somatic diagnoses (1.25; 0.84–1.86). Among significant risk factors for DP in TRD were female sex, being > 29 years of age, unemployment and a diagnosis of comorbid personality disorder (ICD-10 codes F60.0–9).

**Conclusion:**

TRD is associated with an elevated risk for DP compared to other patients with depression, with large potential costs for the affected patients and for society. Clinical and therapeutic implications for patients with TRD who are granted DP should be further investigated. Limitation: No clinical data, e.g. type of depression or reason for treatment switch, was available for this study.

## Background

Depression is a global health problem, affecting about 6% of the population annually worldwide [1]. The lifetime prevalence is estimated at 16% [1]. The risk for onset of depression is highest between the ages of 16–43, with a median age of onset at 25 years [2]. Among adults, depression risk is doubled among women compared to men [3].

By definition, the diagnosis of depression entails significant distress or impairment in social, occupational, or other important areas of functioning [4]. Depression is associated with a number of adverse outcomes for the afflicted individual, such as elevated risk for chronic somatic disease [5], impaired social and physical functioning [6], and death [7]. Furthermore, depression inflicts a large cost on society due to direct and indirect medical costs [8]. This in combination with high incidence, high risk of recurrence, and a substantial proportion of patients being treatment resistant, depression is one of the leading causes of years lost to disability globally, representing 5% of the total burden [9].

Outcome studies on depression often focus on symptomatic, rather than functional outcomes, although these do not necessarily correlate [10]. Functional outcomes focus on an individual’s recovery in areas such as vocational and social functioning rather than symptom resolution, and are normally not included as outcome parameters in clinical trials. Work disability may be one of the most important functional measures for the individual and for society. It can be measured as temporary, leading to sickness absence, or permanent, in which case the individual may be eligible for disability pension (DP) depending on the national insurance system. Expenditures for DP generally make up a considerable percentage of the total costs for social insurance in welfare states such as Sweden [11]. DP may have large economic and social consequences for the individual, including lower lifetime income and poor quality of life [12]. In a recent meta-analysis including 15 cohort studies, depression was found to increase the risk for DP compared to non-depressed subjects with a pooled risk ratio of 1.82 (95% confidence interval [CI] 1.45–2.28) among men and 1.62 (95% CI 1.31–2.02) among women [13].

Treatment-resistant depression (TRD) is most commonly defined as not responding or achieving symptom remission despite two adequate treatment trials [14, 15]. In the second phase of the multicentre European Group for Study of Depression trial, 40% of patients who initiated antidepressant treatment were affected by TRD [16]. TRD has been associated with an elevated risk of death and suicide [17], and with a higher number of recurrent depressive episodes and hospitalizations [18] compared to depression not classified as treatment-resistant.

TRD has been suggested to be a major contributor to the burden of work disability associated with depression [19]. Depression symptom duration correlates with work disability [20] and the length of depressive episodes seems to predict long-term work disability [19, 21]. Of depressed patients on sick leave, one third may not return to work within 1 year [22]. Patients who achieve symptom remission show significantly greater improvement in self-rated work functioning compared to those who respond but do not remit [23]. In one clinical study, TRD out-patients incurred higher costs than other depressed patients related to the number of working days lost, and these costs did not correlate with depression severity [24]. Also, a recent re-analysis of data from the Sequenced Treatment Alternatives to Relieve Depression (STAR*D) trial showed greater functional and work impairment as well as lower work productivity among the patients classified with TRD compared to other depressed patients [25].

In clinical studies, there are few reports on long-term consequences for patients with TRD and no studies which report long-term sickness absence or DP [26]. Neither have risk factors for being granted DP among patients with TRD been reported. Among the known risk factors for being granted DP among patients with depression are being female, below 25 or above 45 years of age, low educational level, living alone, residing outside big cities and being born outside Europe, and DP is also associated with antidepressant medication, psychiatric and somatic comorbidities, and long-term sick leave [27, 28]. In order to fill this gap, pharmacoepidemiological definitions of TRD may be used for research in administrative data. In general, a third claim for an antidepressant drug or other treatment for depression within a specified time frame has been used as a proxy for TRD, an approach that has been used for health economic analyses in US claims data [29–32] as well as in studies in Swedish, British and Taiwanese administrative databases [17, 33, 34].

The aims of this study were (a) to investigate the risk for being granted all-cause and diagnosis-specific DP among patients with TRD compared to other depressed patients with active antidepressant treatment, as a whole and stratified by sex, age, type of living area and history of depression, and (b) to investigate the association between various clinical and sociodemographic factors and DP among patients with TRD.

## Methods

### Data sources and study population

This prospective case-cohort study was performed linking data from a combination of nationwide registers in Sweden. Register data is available for researchers after formal application and with a valid ethical permit. Linkage between registers is made through the unique personal identification number assigned to all residents in Sweden, after which data is anonymized before delivery.

The cohort was identified through registers held by the National Board of Health and Welfare. We identified all patients 18–65 years old with a dispensed prescription of an antidepressant (ATC [Anatomical Therapeutic Chemical]-code N06A), between the years of 2006–2013 in the Prescribed Drug Register (PDR) [35].. The date of the first dispensing in the current episode is hereafter called the index date. The PDR covers data on all dispensed drugs at Swedish pharmacies since July 2005, including drug ATC-code and strength, packet size, date of prescription and dispensing, and also prescriber’s text instruction when issued. To identify new treatment episodes, only those patients were selected who had no dispensing or recording within 180 days before the index date of a) an antidepressant or any potential add-on medication for treatment of depression according to clinical guidelines (antipsychotics, lithium, lamotrigine, valproate or carbamazepine) [36], and b) administration of ECT (electroconvulsive therapy) or rTMS (repetitive transcranial magnetic stimulation) registered in the National Patient Register (NPR). The NPR includes data on all ICD-10 diagnostic and procedure codes registered during health care contacts in specialized (excluding primary) care, with nationwide in- and out-patient coverage from 1987 and 2001, respectively [37]. Diagnoses in the NPR in general have satisfactory to excellent clinical validity, although e.g. depression has not been specifically validated [37]. Any diagnosis, including depression, is registered by a physician in a health practice setting, in which structured diagnostic interviews for confirmation of diagnoses are common but not mandatory. Patients had to have been residents in Sweden during the full 180 days preceding the dispensing year according to the Total Population Register (TPR), which is a census register held by the government agency Statistics Sweden which contains all individuals resident in Sweden, including immigration and emigration dates and limited demographic information [38]. Further, covariates and outcomes listed in following sections were acquired from the Micro-Data for Analyses of Social insurance (MiDAS) register, held by the National Social Insurance Agency, which includes data on dates and diagnoses of sick leave episodes and DP [39], and sociodemographic data obtained from Statistics Sweden.

In order to identify patients with depression as main treatment indication, we selected those patients who had depression as main diagnosis (ICD-10 codes [International Classification of Diseases, 10th version] F32-F33) registered in the NPR within a time interval of 30 days before, and up to 365 days after, the index date. This interval was chosen to capture also patients with initial treatment in primary care which is not covered by the NPR. Patients with any other major psychiatric disorder, defined as a diagnosis of dementia, psychotic or bipolar disorder ever registered in the NPR before the index date, were excluded.

### Definition of TRD

We used a definition of TRD constructed for use in Swedish register data [17]. TRD criteria were met when initiating a third sequential treatment trial for depression after two preceding adequate trials, including the index trial, all within a period of 365 days. The treatment episodes were constructed from data in the PDR regarding dosage, dispensations, package-sizes and prescription texts. An adequate treatment episode was defined as lasting for at least 28 days, and for antidepressants this had to be at least at the lowest defined daily dose for depression according to the ATC-code of the drug. No longer gaps than 28 days were allowed between episodes in order to emulate sequential treatment. Second and third treatment episodes could consist of a) new antidepressant treatment, b) add-on medication with lithium, antipsychotics or anticonvulsants to an existing antidepressant, or c) series of ECT or rTMS. This identified 4377 patients with TRD, of whom 3427 were not on DP at the index date and hence included in the study.

### Selection of comparators

As many factors may affect the association between TRD and DP, we aimed for close socio-demographic and clinical matching between cases and comparators. At the date when TRD criteria were met, each patient with TRD was matched with one comparator - who was not on DP - from the remainder of the depression cohort according to a) age at year of index date (±3 years), b) sex, c) calendar year of the index prescription, d) type of living area (classified as larger cities, medium-sized municipalities or smaller municipalities), and e) history of a depression diagnosis 1–5 years before index date. The latter was chosen as matching criterion as we hypothesized that it would have substantial impact on the trajectory of antidepressant treatment trials. In order to minimize follow-up bias, comparators also had to have an ongoing 1st or 2nd treatment episode (defined as above through package size and dosing text) to be available for selection. No matching comparators could be found for 223 of the patients with TRD, and they were hence excluded from the study. Cases without comparators were more likely to be male (54.4% vs. 42.2% of cases with comparators) and older (median age 44, IQR 32–56 vs. 36, IQR 25–48) than TRD cases with comparators.

The final sample included *N* = 3204 patients with TRD and N = 3204 comparators.

### Outcomes

In Sweden, residents aged 19–64 years with a reduced work capacity due to disease or injury can be granted full or part-time DP by the Social Insurance Agency. People aged 19–29 years can be granted temporary DP not only due to work incapacity but also to complete upper-secondary education, and individuals 30–64 years of age can be granted permanent DP. Approximately 1% of the Swedish population of working age receives DP [11]. The main outcome was granting of DP, part- or full-time, due to any main cause as stated in the MiDAS register.. Secondary outcomes were the first registered occurrence of being granted DP with the main cause diagnosis of depression (ICD-10: F32–33), any mental and behavioural disorder (F00–99), or somatic disorder (all causes, except for F00–99).

### Covariates

The analysis also included multiple clinical and sociodemographic covariates. Clinical covariates, measured as diagnoses in in- or outpatient care < 5 years before index date, were: a) history of anxiety disorders (ICD-10: F40-F41), b) history of personality disorders (F60-F61), c) history of other major psychiatric disorder (obsessive compulsive disorders [OCD] F42, eating disorder F50, autism, and hyperactivity disorder F84.0–1, F84.5, F90), d) history of self-harm/suicide attempt (X60-X84, as well as Y10-Y34 [harm with undetermined intent] to avoid underreporting), and e) somatic disorders, expressed as Charlson Comorbidity Index [40] constructed from diagnostic data in the NPR and categorized as 0, 1 and ≥ 2 major comorbidities. In addition, f), substance use disorders (SUD), were categorized in two mutually exclusive categories, alcohol use disorder only, and other/mixed SUD. Alcohol use disorder only included patient with recorded diagnoses of F10.1-F10.9 and/or prescription of disulfiram N07BB01, acamprosate N07BB03, naltrexone N07BB04 or nalmefen N07BB05), but not F11–16, F18–19 or prescription of sublingual buprenorphine N07BC01, N07BC51 or methadone N07BC02. If any of the latter diagnoses or prescriptions were present, the patient was counted as other/mixed SUD.

The analyses also included sociodemographic covariates from Statistics Sweden, using the last available data at the index date: a) attained education level (< 9 years, 10–12 years, > 12 years), b) family situation (married/co-habiting, living without children; married/co-habiting, living with children; single, living without children; single, living with children; aged ≤20 years and living with parent/−s), c) country of birth (Sweden/other Europe/other than Europe). In addition, we assessed previous long-term sickness absence (more than 90 net days on sickness absence during ≤365 days before index date), and employment status during the calendar year preceding the year of index date (0, 1–179 days, or ≥ 180 days of registered unemployment).

### Statistical analysis

Complete data were available for the years 2006–2013, which constituted the follow-up period. The follow-up started at the date of fulfilment of TRD definition for patients with TRD and at the corresponding matching date for non-TRD comparators. If a comparator was defined with TRD during the follow-up (i.e. moved from comparator group to TRD group), the follow-up as comparator was censored. The follow-up ended at DP, death, emigration, diagnoses of schizophrenia or bipolar disorder, the end of study follow-up (December 31st, 2013) or if a comparator fulfilled the TRD definition, whichever came first. Proportional Cox regression with TRD status as time-dependent exposure was applied, taking the matched design into account by conditioning on (separate strata) each matched group. All adjusted analyses included all clinical and sociodemographic covariates described above. The main outcome was DP due to any cause, calculating crude and adjusted hazard ratios (aHR) with 95% confidence intervals (CI) comparing patients with TRD and comparators. For sensitivity, the analysis was also performed stratified on the matching variables. Analyses were also performed with regard to diagnosis-specific DP: due to depressive disorders, any mental or behavioural disorder, and somatic disorders. Risk for DP among patients with TRD compared with comparators was also assessed stratified by each category of matching criteria. Lastly, all covariates were analysed as risk factors for DP separately among patients with TRD. Both unadjusted and adjusted analyses were performed. In the adjusted models all covariates listed above were analysed simultaneously and no variable selection driven from material was performed. Statistical analyses were performed with SAS version 9.3 (SAS Institute, Inc., Cary, NC, USA).

## Results

Table 1 shows descriptive data of the study cohort, which included 3204 persons with TRD and 3204 matched comparators with depression. The majority were women (58%) and mean age was 37 years (±SD 13). Median follow-up time in the study was somewhat shorter in the TRD cohort than among comparators (1505 days, IQR 494–2105, vs. 1797 days, IQR 669–2238).

**Table 1:** Characteristics of patients with treatment resistant depression (TRD) vs matched comparators^a^ with treatment for depression

	TRD *N* = 3204	Comparators *N* = 3204	*p*
n (%)			
**Men**	1363 (42.5)	1363 (42.5)	matched
**Women**	1841 (57.5)	1841 (57.5)	
**Age (years)**^b^			
18–29	1245 (38.9)	1251 (39.0)	matched
30–49	1333 (41.6)	1328 (41.5)	
50–69	626 (19.5)	625 (19.5)	
**Type of living area**			matched
Small municipality	842 (26.3)	842 (26.3)	
Medium-sized municipality	1109 (34.6)	1109 (34.6)	
Large city	1253 (39.1)	1253 (39.1)	
**Attained education level**^b^			**0.0017**
≤ 9 years	969 (30.2)	842 (26.3)	
10–12 years	1442 (45.0)	1504 (46.9)	
≥ 13 years	793 (24.8)	858 (26.8)	
**Family situation**^b^			0.0942
Married, living without children	296 (9.2)	310 (9.7)	
Married, living with children	768 (24.0)	695 (21.7)	
Unmarried, living without children	1419 (44.3)	1508 (47.1)	
Unmarried, living with children	327 (10.2)	299 (9.3)	
≤ 20 years, living with parent/−s	394 (12.3)	392 (12.2)	
**Country of birth**			**< 0.0001**
Sweden	2420 (75.5)	2647 (82.6)	
Other European country	165 (5.2)	123 (3.8)	
Other than Europe	619 (19.3)	434 (13.6)	
**Long-term sickness absence**^c^	429 (13.4)	418 (13.1)	0.6849
**Unemployment**^**d**^			0.2803
1–179 days	643 (20.1)	620 (19.4)	
≥ 180 days	185 (5.8)	161 (5.0)	
**History of depression diagnosis**	536 (16.7)	536 (16.7)	matched
**History of anxiety disorder**^e^	621 (19.4)	638 (19.9)	0.5930
**History of personality disorder**^e^	97 (3.0)	87(2.7)	0.4544
**History of substance use disorder**^e^			**0.0425**
None	2895 (90.4)	2847 (88.9)	
Alcohol only	143 (4.5)	187 (5.8)	
Other/mixed	166 (5.2)	170 (5.3)	
**History of other psychiatric disorder**^e^	190 (5.9)	195 (6.1)	0.7927
**History of self harm/suicide attempt**^e^	208 (6.5)	277 (7.1)	0.3454
**CCI**^e, f^			0.7685
0	3029 (94.5)	3016 (94.1)	
1	143 (4.5)	155 (4.8)	
≥ 2	32 (1.0)	32 (1.0)	

^a^Comparators matched on age, sex, calendar year of the index prescription, history of depression, and size of living area

^b^At time of first antidepressant prescription

^c^ > 90 net days in the 365 days preceding first antidepressant prescription

^d^In the year preceding first antidepressant prescription

^e^Within 5 years before first antidepressant prescription

^f^Charlson’s comorbidity index [40]

**Fig. 1:**
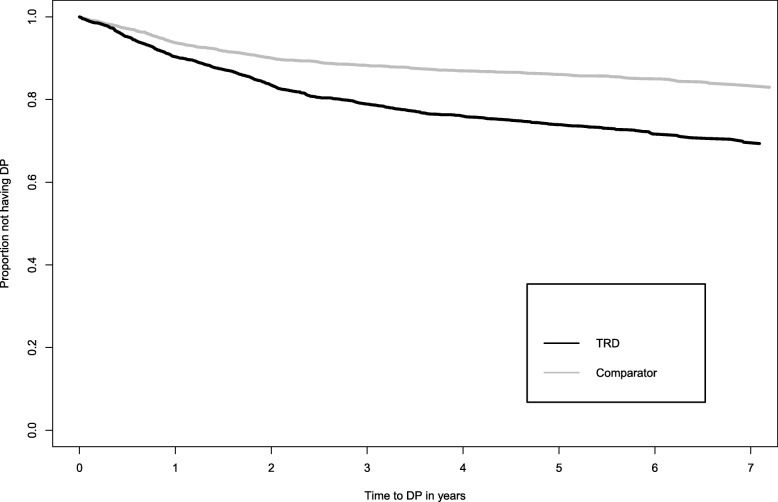
Time to disability pension (DP) due to any cause, comparing cases with treatment resistant depression (TRD) and matched comparators with treatment for depression

During follow-up, 730 patients with TRD and 406 comparators with depression were granted DP, representing incidence rates of DP per 100 persons-years of 6.16 (95% CI 6.12–6.21) and 3.03 (3.00–3.06), respectively. Crude and adjusted HRs from the full model are shown in Table 2, with the corresponding Kaplan-Meier Curves in Fig. 1. TRD was associated with a doubled risk for all-cause DP compared to comparators (aHR 2.07, 95% CI 1.83–2.35). The effect was significant over the matching strata (Table 3).

**Table 2:** Risk for disability pension (DP) among patients with treatment-resistant depression (TRD) vs. matched comparators with treatment for depression. Unadjusted and adjusted hazard ratios (HR) with 95% confidence intervals (CI) for all-cause and cause-specific DP

Outcome	N (% of DPs granted)	Unadjusted HR (95% CI)	Adjusted HR (95% CI)^**b**^
**All-cause DP**			
Comparators^**a**^ (n = 3204)	406 (100)	Ref	Ref
TRD (n = 3204)	730 (100)	1.90 (1.72–2.11)	**2.07 (1.83–2.35)**
**DP due to mental and behavioural disorders**			
Comparators	309 (76.1)	Ref	Ref
TRD	621 (85.1)	2.13 (1.89–2.39)	**2.24 (1.95–2.57)**
*Of which DP due to depression (F32–33)*			
*Comparators*	134 (43.4)	Ref	Ref
*TRD*	277 (44.6)	2.15 (1.80–2.56)	**2.28 (1.82–2.85)**
**DP due to somatic disorders**			
Comparators	97 (23.9)	Ref	Ref
TRD	109 (14.9)	1.22 (0.98–1.51)	1.25 (0.84–1.86)

^a^Comparators matched on age, sex, calendar year of the index prescription, history of depression, and size of living area

^b^Adjusted for attained educational level, marital/ parental status, country of birth, long-term sickness absence, unemployment, anxiety, personality and other psychiatric disorders, substance use disorder, history of self-harm/suicide attempt and Charlson’s Comorbidity index

**Table 3:** Risk for all-cause disability pension (DP) among patients with treatment-resistant depression (TRD) vs. matched comparators with treatment for depression, stratified by matching factors. Unadjusted and adjusted hazard ratios (HR) with 95% confidence intervals (CI)

	Unadjusted HR (95% CI)	Adjusted HR (95% CI)^a^
**All**	1.90 (1.72–2.11)	**2.07 (1.83–2.35)**
**Sex**		
Men	1.77 (1.52–2.08)	**1.84 (1.50–2.24)**
Women	2.00 (1.75–2.29)	**2.29 (1.94–2.71)**
**Age (years)**^b^		
18–29	2.31 (1.90–2.82)	**2.50 (1.97–3.16)**
30–49	1.80 (1.54–2.10)	**2.05 (1.68–2.50)**
50–69	1.61 (1.32–1.97)	**1.85 (1.39–2.46)**
**Type of living area**		
Small municipality	2.03 (1.66–2.47)	**2.41 (1.83–3.17)**
Medium-sized municipality	1.49 (1.26–1.76)	**1.43 (1.13–1.80)**
Large city	2.27 (1.91–2.70)	**2.62 (2.10–3.28)**
**History of depression diagnosis**^c^		
Yes	1.78 (1.39–2.28)	**2.12 (1.50–2.99)**
No	1.93 (1.72–2.16)	**2.13 (1.85–2.45)**

^a^Adjusted for attained educational level, marital/ parental status, country of birth, long-term sickness absence, unemployment, anxiety disorders, personality disorders, and other psychiatric disorders, substance use disorder, history of self-harm/suicide attempt and Charlson’s Comorbidity index

^b^At time of first antidepressant prescription

^c^ICD-10 diagnosis F32-F33

As seen in Table 2, a higher proportion in the TRD group was granted DP due to mental disorders than among comparators (85% vs 76%). DP due to depressive disorders were 45 and 43% of all DPs granted, respectively. The most common diagnostic subclasses of DPs due to mental and behavioural disorders were mood disorders (ICD-10 F3, 51.5%) and neurotic, stress-related and somatoform disorders (ICD-10 F4, 25.1%; all frequencies available in Supplemental Table 1). TRD was associated with a significantly increased risk of DP due to depressive disorders (aHR 2.28; 95% CI 1.82–2.85) and DP due to any mental and behavioural disorder (aHR 2.24, 95% CI 1.95–2.57) but not due to somatic disorders (aHR 1.25, 95% CI 0.84–1.86)..

In the risk factor analysis for DP among patients with TRD (Table 4), significant sociodemographic risk factors were: being female compared to male (aHR 1.21, 95% CI 1.03–1.41), older age compared to 18–29 years old (50–69 years old: aHR 1.70, 95% CI 1.32–2.19), being born outside of Europe compared to in Sweden (aHR 1.55, 95%CI 1.29–1.85), long-term sickness absence (aHR 3.26, 95% CI 2.74–3.87) and being unemployed (aHR 1.24, 95%CI 1.03–1.50). Regarding family status, being (≤20y) and living with parents inferred the highest risk (aHR 1.90, 95% CI 1.30–2.78) and living in a single household with children the lowest risk (aHR 0.66, 95%CI 0.46–0.94), compared to being married/cohabitant without children (ref). Significant clinical risk factors were having a diagnosis of personality disorder (aHR 1.58, 95%CI 1.05–2.38) and CCI 1 or above (CCI 1: aHR 1.41, 95% CI 1.03–1.92).

**Table 4:** Risk factors for disability pension (DP) among patients with treatment-resistant depression (TRD). Unadjusted and adjusted hazard ratios (HR) with 95% confidence intervals (CI) for all-cause DP

	Unadjusted HR (95% CI)	Adjusted HR (95% CI)^**a**^
**Sex**		
Men	Ref	Ref
Women	1.11 (0.95–1.28)	**1.21 (1.03–1.41)**
**Age (years)**^**b**^		
18–29	Ref	Ref
30–49	1.28 (1.08–1.52)	**1.32 (1.05–1.66)**
50–69	1.63 (1.34–1.98)	**1.70 (1.32–2.19)**
**Size of living area**		
Small municipality	1.01 (0.85–1.21)	1.08 (0.90–1.29)
Medium-sized municipality	0.85 (0.72–1.01)	0.89 (0.75–1.07)
Large city	Ref	Ref
**Attained education level**^**b**^		
≤ 9 years	1.20 (0.98–1.46)	1.20 (0.97–1.49)
10–12 years	1.12 (0.93–1.35)	1.09 (0.90–1.32)
≥ 13 years	Ref	Ref
**Family situation**^**b**^		
Married, living without children	Ref	Ref
Married, living with children	0.96 (0.75–1.24)	1.03 (0.78–1.36)
Unmarried, living without children	0.75 (0.59–0.96)	1.03 (0.79–1.34)
Unmarried, living with children	0.64 (0.46–0.89)	**0.66 (0.46–0.94)**
≤ 20 years, living with parent/−s	0.93 (0.69–1.25)	**1.90 (1.30–2.78)**
**Country of birth**		
Sweden	Ref	Ref
Other European country	1.30 (0.96–1.77)	1.32 (0.96–1.80)
Other than Europe	1.59 (1.35–1.88)	**1.55 (1.29–1.85)**
**Long-term sickness absence**^c^		
No	Ref	Ref
Yes	3.17 (2.70–3.72)	**3.26 (2.74–3.87)**
**Unemployment**^d^		
None	Ref	Ref
1–179 days	1.04 (0.87–1.25)	**1.24 (1.03–1.50)**
≥ 180 days	1.11 (0.82–1.50)	1.24 (0.91–1.69)
**History of depression**		
No	Ref	Ref
Yes	1.06 (0.87–1.30)	1.01 (0.82–1.25)
**History of anxiety disorder**^e^		
No	Ref	Ref
Yes	0.92 (0.76–1.12)	0.91 (0.75–1.12)
**History of personality disorder**^e^		
No	Ref	Ref
Yes	1.46 (0.99–2.16)	**1.58 (1.05–2.38)**
**Substance use disorder**^e^		
None	Ref	Ref
Alcohol only	0.73 (0.47–1.12)	0.74 (0.47–1.15)
Other/mixed	0.82 (0.56–1.21)	0.72 (0.48–1.07)
**History of other psychiatric disorder**^e^		
No	Ref	Ref
Yes	1.12 (0.81–1.54)	1.26 (0.90–1.78)
**History of self harm/suicide attempt**^e^		
No	Ref	Ref
Yes	1.15 (0.86–1.55)	1.19 (0.87–1.63)
**CCI**^5, 6^		
0	Ref	Ref
1	1.60 (1.18–2.17)	**1.41 (1.03–1.92)**
≥ 2	1.34 (0.69–2.59)	0.87 (0.45–1.69)

^a^Adjusted for all covariates

^b^At time of first antidepressant prescription

^c^ > 90 net days in the 365 days preceding first antidepressant prescription

^d^In the year preceding first antidepressant prescription

^e^Within 5 years before first antidepressant prescription

^f^Charlson’s comorbidity index [40]

## Discussion

The main finding of this study was that patients with TRD had a doubled risk for being granted DP compared to other patients with depression and ongoing treatment. The risk was similar among patients with a first-time depression diagnosis compared to those with recurrent depression. The risk was increased for DP granted due to a depressive disorder or any mental disorder, but not for somatic diagnoses. Among patients with TRD, being female, of older age, unemployed, born outside Europe and having a history of personality disorder were risk factors for DP.

### Association between TRD and DP

Results from the present study illustrate that patients fulfilling our definition of TRD – i.e. patients initiating a third treatment trial for depression - had a substantial risk increase for DP compared with patients with an ongoing first or second treatment. A possible confounding mechanism behind the association between TRD and DP is that work disability itself is a risk factor for depression, and patients with work disability may receive more active treatment interventions from clinicians compared to those with no work disability which would lead to easier fulfilling our definition of TRD [41]. However, results were still significant after adjustment for previous long-term sickness absence. Likewise, patients actively seeking DP may have more health care contacts and hence more opportunities for treatment trials introducing follow-up bias; the matching criteria of active treatment among the comparators was meant to address this issue.

The relationship between depression, TRD and DP is bound to be complex. Comorbidity of somatic and psychiatric disorders may induce depression and treatment resistance, as well as elevate the risk for DP [42]. Depression and the risk for TRD may both be related to personality traits such as negative valence and neuroticism [43], which in turn may be independent risk factors for disability pension [44]. Factors associated with both TRD and DP may contribute to this association. Patients with TRD are more likely to spend more time in a depressed state, which contributes to the risk for DP from depression [28]. In addition, patients defined with TRD may actually be experiencing so called “pseudo-resistance” due to undiagnosed psychotic or bipolar disorders, which carry a high risk for DP [45–47].

The Kaplan-Meier curves showed the highest deviance in granted DP between TRD and comparators patients during 500–1000 days after start of follow-up and the difference remained to the end of follow-up. This finding may be of importance as it shows that the TRD definition divides patients into different risk groups years before DP is granted, which is consistent with this decision process often taking several years.

A large proportion of patients with TRD, and to a lesser degree, comparators, were granted DP during follow-up, and most had been neither on long-term sick leave (13%) nor unemployed (25%) at antidepressant treatment initiation (although numbers were markedly higher than the approximately 6% estimated for both measures in the general population in Sweden [48, 49]). This may further illustrate the potential impact of both MDD and TRD on risk for DP, but should also be viewed in light of a significant proportion of the cohort being < 30 years of age, and many in this age group could be in the educational system or work apprenticeship, and hence less included in these social insurance systems.

### Risk factors for DP among patients with TRD

Among the patients with TRD, women were at higher risk for DP than men, corresponding to the known elevated risk for DP among women in general [50]. As women are also at higher risk for both depression and for subsequent TRD [51], further investigation of these mechanisms seems highly prioritized considering that there are inequities regarding both mental health efforts [52] and disability pension risk [53] towards women.

Risk for DP increased with age, also in line with the existing literature [54]. Being born outside of Europe is another established risk factor, which may be attributed to adjustment difficulties to a new societal context and labour market and perhaps cultural differences regarding acceptance of mental distress and occupational function [54]. Regarding socio-economic status, lower attained education level did not increase risk for DP significantly compared to higher in this study, in contrast with existing literature on the impact of education in general [55]. This may reflect that the occupational disability due to TRD may be equally prominent among individuals normally at lower risk for DP such as academics. We did not include income level as a socio-economic measure in the present study, which would an interesting factor to investigate to see if the impact of TRD also attenuates the known association between low income and TRD [54]. Regarding family situation, patients in single households with children surprisingly had lower risk for DP than married/cohabitants without children. This may be related to that the substantial drop in income after DP may be a motivation factor to stay in employment as long as necessary in a perhaps already stressed economic situation. Patients < 20 years living at home had highest risk for DP in the adjusted, but not crude, analysis, which may be related to the granting of temporary DP discussed above.

Regarding clinical risk factors, somewhat unexpectedly a history of depressive episodes did not increase the risk for DP. Recurrent depression is a known risk factor for TRD [56], but if results here are to believed, it is the TRD, not recurrence, that has an impact on risk for DP although it has been previously demonstrated that time spent in depression may increase risk for DP [28]. A history of suicide attempts/self-harm may be related to both severity of depression and risk for TRD, but did likewise not emerge significant in this study [16].

History of SUD and anxiety disorders did not affect risk for DP among the patients in this study. While these comorbidities may increase risk for DP in themselves, there has been little additional effect demonstrated when present as comorbid conditions in depression [28]. Personality disorders and somatic comorbidity, however, have been reported to increase risk for DP in patients with depression and emerged as significant risk factors in this study [28, 57].

### Strengths and weaknesses

Strengths of this study include the use of nationwide Swedish registers of high quality and coverage, allowing adjustment for clinical and sociodemographic covariates, a relatively long follow-up time with no loss of follow-up. The matching requirement of ongoing treatment in the comparator cohort lowered the risk for follow-up bias. Also, we included only patients with a depression diagnosis from specialized healthcare, increasing the validity of the diagnosis.

Weaknesses of this study are also, however, related to the register-based setting. The definition of TRD used here is a proxy model, with unknown validity in comparison to clinical evaluation although similar models have been used in various data sources previously in Sweden and other countries [17, 29, 33] . Although clinical accuracy of the NPR is generally high, the diagnosis of depression has not yet not been validated, and validation of the corresponding register in the similar country of Denmark showed moderate precision [58]. Clinical data on severity of depression was not available. Reasons for continuing or discontinuing treatment are unknown, and may be related to adverse reactions, improvement of symptoms or loss to clinical follow-up.

### Implications

While this study is set within the Swedish health care and social insurance system, the results should be generalizable to other countries with similar health care and social insurance regulations. In Sweden, DP is granted when the work capacity loss is considered long-term (for patients < 30 years old) or permanent by both the physician and by the Social Insurance Agency, which should equal the highest level of work disability. Considering the high prevalence of both depression, and eventual high rates of TRD among those initiating treatment for depression, this may infer vast economic consequences for the society, as well as a large, long-term or permanent income loss for the patient. Results from this study highlight the need for measures that prevent or improve TRD status to avoid these costs, as well as for rehabilitating measures to preserve or improve occupational functioning among patients with TRD. The fact that over a third of patients in this study were under thirty years of age and that these had the highest risk for DP compared to other depressed patients may be of special concern, especially in the context of the often early age of onset of depression [59] and that depression onset in younger age has been demonstrated to increase risk for TRD [60].

## Conclusion

Patients with TRD are at higher risk for being granted DP than other patients treated for depression. Prevention of TRD status and contributing mechanisms may decrease risk for DP. The costs for society and the individual due to TRD-related work disability should be further investigated.

## Supplementary information

**Additional file 1: Supplementary Table 1.** Frequencies of persons granted with disability pension (DP) due to specific mental and behavioural disorders, compared between persons with treatment resistant depression (TRD) and comparators.

## Data Availability

The data that support the findings of this study are available from the Swedish government agencies National Board of Health and Welfare, and Statistics Sweden, but restrictions apply to the availability of these data, which were used under license for the current study and so are not publicly available.

## Abbreviations

aHR: Adjusted hazard ratio
ATC: Anatomical Therapeutic Chemical
CI: Confidence interval
DP: Disability pension
ECT: Electroconvulsive therapy
ICD-10: International Classification of Diseases, 10th version
MDD: Major depressive disorder
MiDAS: Micro-Data for Analyses of Social insurance
NPR: National Patient Register
OCD: Obsessive compulsive disorder
rTMS: Repetitive transcranial magnetic stimulation
STAR*D: Sequenced Treatment Alternatives to Relieve Depression
SUD: Substance use disorders
TPR: Total Population Register
TRD: Treatment resistant depression
