# Supplementary material for: Risk and risk factors for disability pension among patients with treatment resistant depression– a matched cohort study
Source: BMC Psychiatry. 2020 May 13;20:232. doi: 10.1186/s12888-020-02642-9 (PMC7222563; doi:10.1186/s12888-020-02642-9)
Supplement: Supplementary file 1 — Additional file 1: Supplementary Table 1. Frequencies of persons granted with disability pension (DP) due to specific mental and behavioural disorders, compared between persons with treatment resistant depression (TRD) and comparators. [file 12888_2020_2642_MOESM1_ESM.docx]

| **Supplementary Table 1. Frequencies of persons granted with disability pension (DP) due to specific mental and behavioural disorders, compared between persons with treatment resistant depression (TRD) and comparators.** | | |
| --- | --- | --- |
| **Sub-classes of all mental and behavioural disorders** | **Comparators**  **N (% of all F diagnoses 309)** | **TRD**  **N (% of all F diagnoses 621)** |
|  |  |  |
| F0 (Organic, including symptomatic, mental disorders) | 6 (1.9) | 12 (1.9) |
| F1 (Mental and behavioural disorders due to psychoactive substance abuse) | 2 (0.7) | 3 (0.5) |
| F2 (Schizophrenia, schizotypal and delusional disorders) | 3 (1.0) | 7 (1.1) |
| F3 (Mood disorders) | 146 (47.3) | 310 (49.9) |
| F4 (Neurotic, stress-related and somatoform disorders) | 70 (22.7) | 167 (26.9) |
| F5 (Behavioural syndromes associated with physiological disturbances and physical factors) | 3 (1.0) | 15 (2.4) |
| F6 (Disorders of adult personality and behaviour) | 29 (9.4) | 52 (8.4) |
| F7 (Mental retardation) | 1 (0.3) | 2 (0.3) |
| F8 (Disorders of psychological development) | 19 (6.2) | 31 (5.0) |
| F9 (Behavioural and emotional disorders with onset usually occurring in childhood and adolescence, and unspecified mental disorder) | 30 (9.7) | 22 (3.5) |
